# Chronic Lymphoproliferative Disorder of Natural Killer Cells: A Rare Event

**DOI:** 10.7759/cureus.10353

**Published:** 2020-09-10

**Authors:** Shafain Sheikh, Sehreen Jahangir, Samreen Khan, Imran Ahmed

**Affiliations:** 1 Immunology, Shifa International Hospital, Islamabad, PAK; 2 Hematology, Shifa International Hospital, Islamabad, PAK

**Keywords:** natural killer cells, clpd-nk, large granular leukemias, aggressive natural killer cell leukemias

## Abstract

Chronic lymphoproliferative disorders are a diverse group of diseases derived from thymus lymphocytes (T cells), bursa of Fabricius cells (B cells), or natural killer (NK) cells. The diagnosis of chronic lymphoproliferative disorders of NK cells (CLPD-NK) is confirmed using antibody panels that are able to detect various stages of maturation of malignant cells. Autoimmune diseases and viral infections are often associated with an increase in circulating NK cells. It is hypothesized that certain viruses trigger the activation of NK cells which leads to the formation of NK cell clones. Majority of the cases are asymptomatic. However, some patients have systemic symptoms and cytopenias. Here, we report a case of CLPD-NK. Our patient’s history and marked lymphocytosis on peripheral film raised the suspicion of a hematolymphoid malignancy for which flow cytometric analysis was done using an extensive panel which confirmed the diagnosis of CLPD-NK.

## Introduction

Chronic lymphoproliferative disorder of natural killer cells (CLPD‐NK) is among the rare group of NK cell malignancies and only a few case reports and small cohorts have been published, this being the first case report from Pakistan. It is characterized by an increase in NK cell lymphocytes in peripheral blood i.e, ≥2 × 10^9^/L for a period of at least six months, without a clearly identified cause [1]. CLPD‐NK can be categorized into three subtypes - the most common being cluster of differentiation (CD) 56 negative/dim and CD16 high NK cell subtype. The next most common is CD56 dim/CD16 negative NK cell subtype, followed by CD56 high/CD16 negative NK cell subtype. Favourable results have been observed with early diagnosis and prompt treatment, but diagnosis is often delayed owing to the fact that NK cells lack a uniquely rearranged antigen receptor gene which makes it difficult to distinguish malignancy from reactive processes [2].

In 2008, the World Health Organization (WHO) has classified CLPD-NK as one of the three categories of large granular leukemias (LGL); the other two being T-cell large granular lymphocytic leukemia (T-LGL), and aggressive natural killer cell leukemia (ANKL) [3]. CLPD-NK has been considered as a provisional entity. It is important to discriminate between ANKL and CLPD-NK as the former is an Epstein-Barr virus (EBV) associated tumor with a poor clinical outcome as opposed to the latter entity.

Limited evidence is available on CLPD-NK which includes a cohort study published in 2014 consisting of 70 patients [4]. Here we report a case of chronic lymphocytosis which was diagnosed as CLPD-NK after extensive immunophenotypic analysis by flow cytometry.

## Case presentation

**Figure 1:**
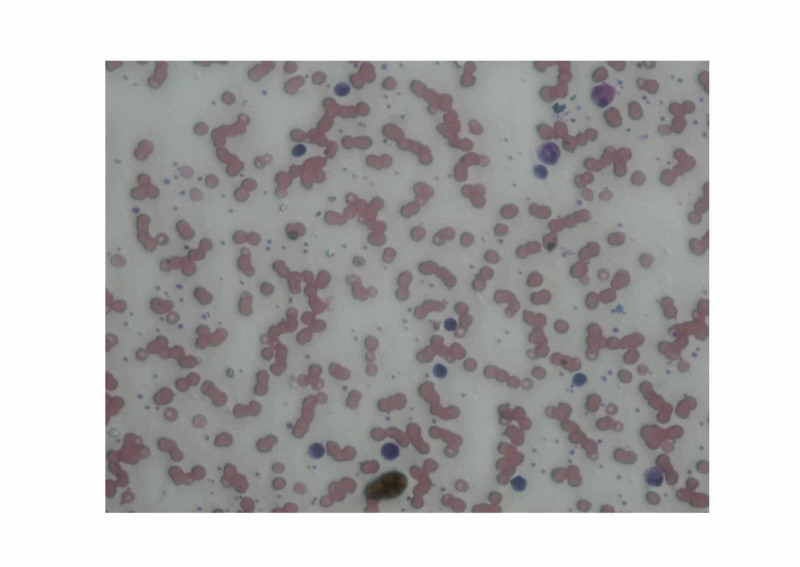
Peripheral film shows normochromic normocytic picture with few reactive lymphocytes

A 38-year-old male presented to the clinic with a complaint of recurrent pilonidal abscesses. His complete blood count revealed leukocytosis (18700/L) with 64% lymphocytes. Peripheral smear is shown in Figure 1. The patient reported a similar presentation two years back. A strong family history of cancers, recurrent abscesses, and persistent lymphocytosis necessitated the need for flow cytometric analysis to investigate for possible hematological malignancy.

Flow cytometric analysis

Flow cytometric immunophenotyping was done on peripheral blood. Lymphoma panel was applied consisting of a total of five tubes having the following panel of monoclonal antibodies with fluorescein isothiocyanate (FITC), phycoerythrin (PE), peridinin chlorophyll protein complex (PerCP), allophycocyanin (APC), and allophycocyanin-cyanine7 (APC-CY7) as flourochromes:

1) 5F/10PE/19PerCP/45APC-CY7

2) Kappa-F/Lambda-PE/19PerCP/20PE-CY7/45APC-CY7

3) FMC-7/23PE/19PerCP/38PE-CY7/11cAPC/45APC-CY7

4) 3F/16+56PE

5) 3F/16+56PE/2PE-CY7/45APC-CY7

**Figure 2:**
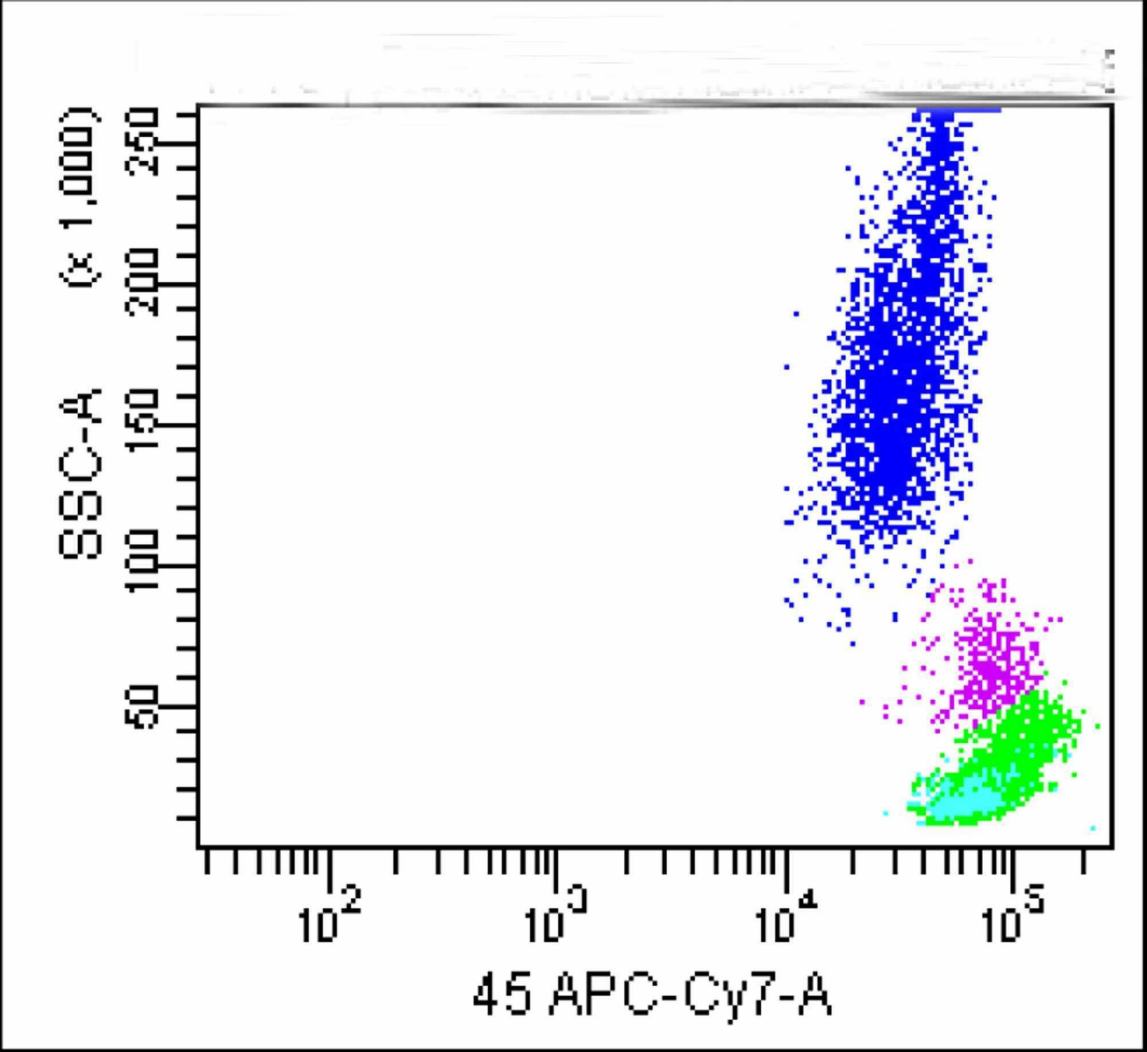
Dot plot shows three cell populations; green population depicts lymphocytes

**Figure 3:**
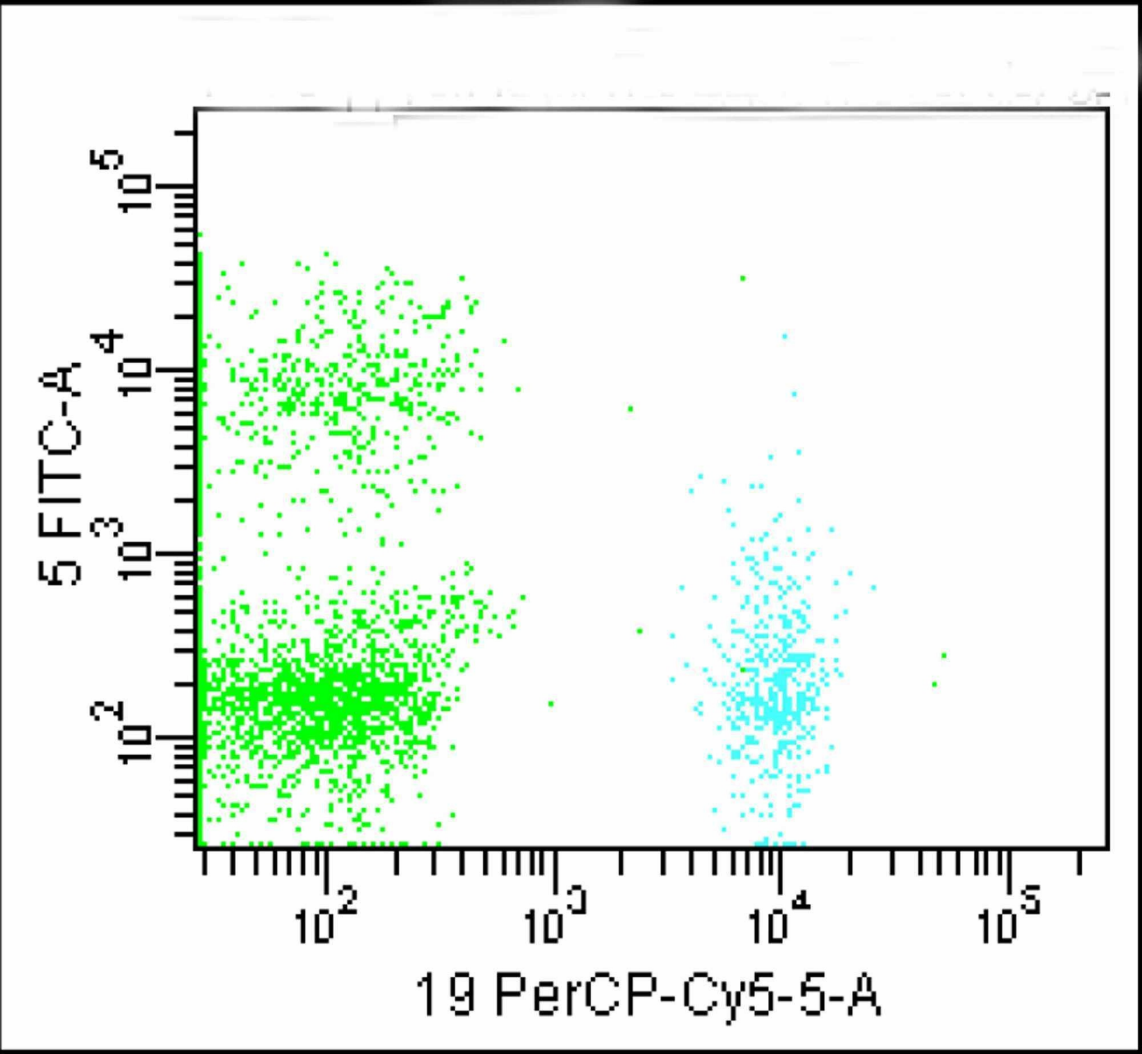
B-lymphocytes (cyan) and T-lymphocytes (upper green population) is identified; the larger green population is negative for CD5 and CD19

A total of 10,000 events were acquired using FACSCanto flow cytometer with FACS Diva software (Becton Dickinson, San Jose, CA). It revealed three distinct cell populations. Of all these, the largest population which appeared to be of lymphocytes was gated with CD 5 Vs CD19 (Figures 2-3).

**Figure 4:**
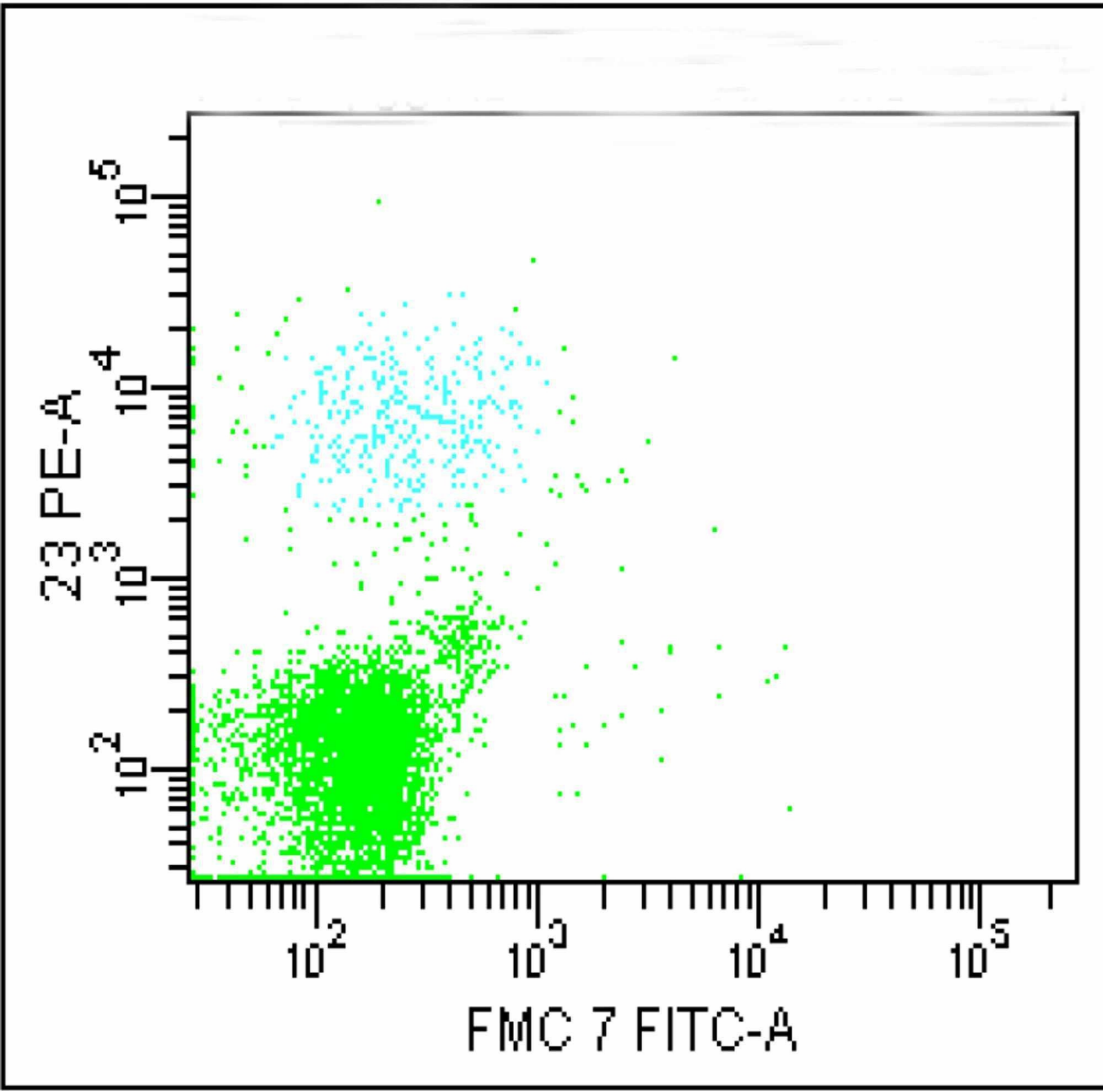
FMC 7 vs. CD23 gating

**Figure 5:**
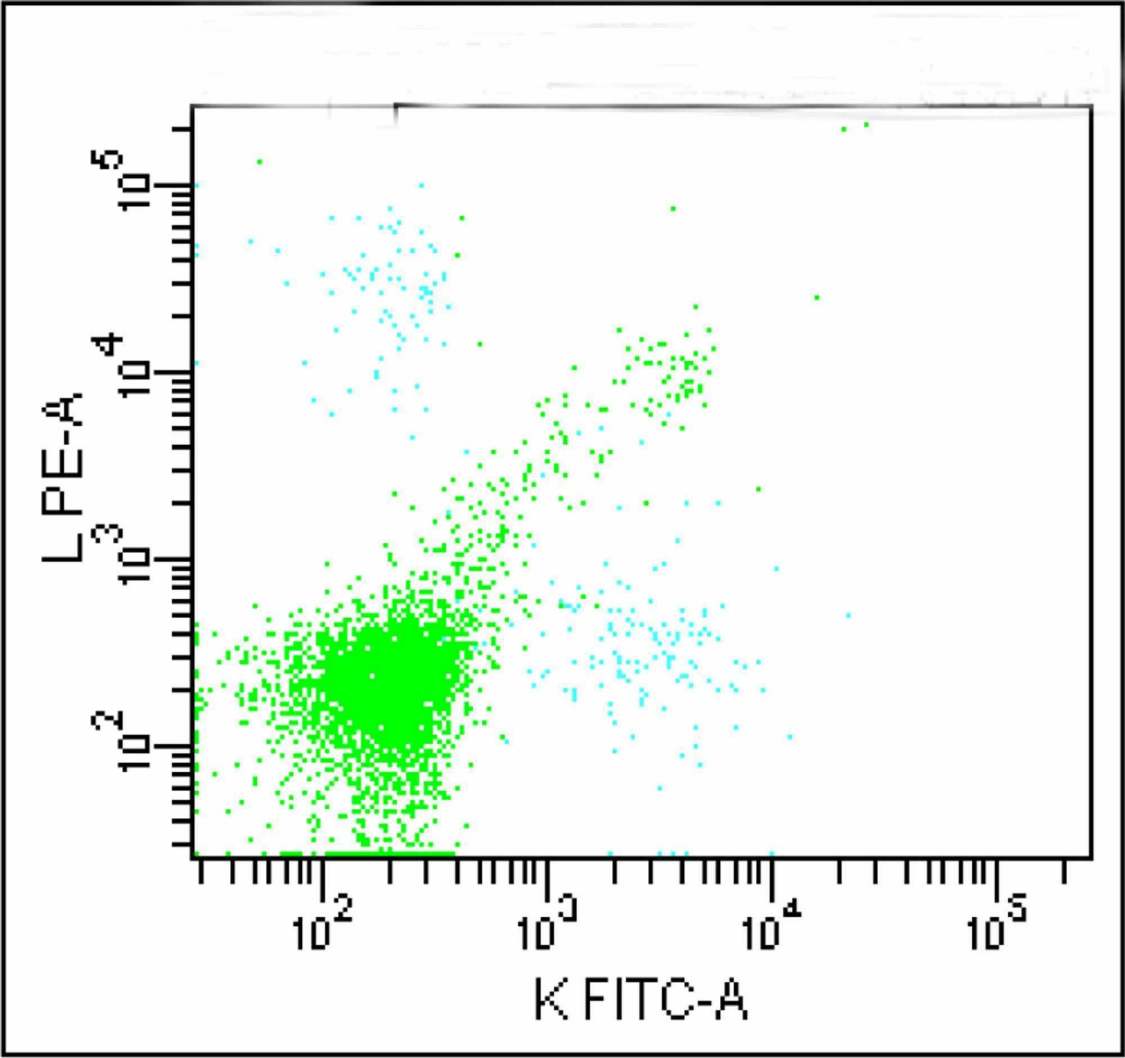
B lypmohocytes (cyan coloured) proportionally distributed for kappa and lambda

This segregated these cells into three distinct populations. A significantly large population of cells (green colored) which was negative for both CD19 and CD5 neither belonged to B or T lymphocyte groups. FMC 7 versus CD23 gating ruled out mantle cell lymphoma and non-Hodgkin lymphoma (Figure 4). Monoclonality was precluded as kappa and lambda showed normal distribution of plasma cells (Figure 5).

**Figure 6:**
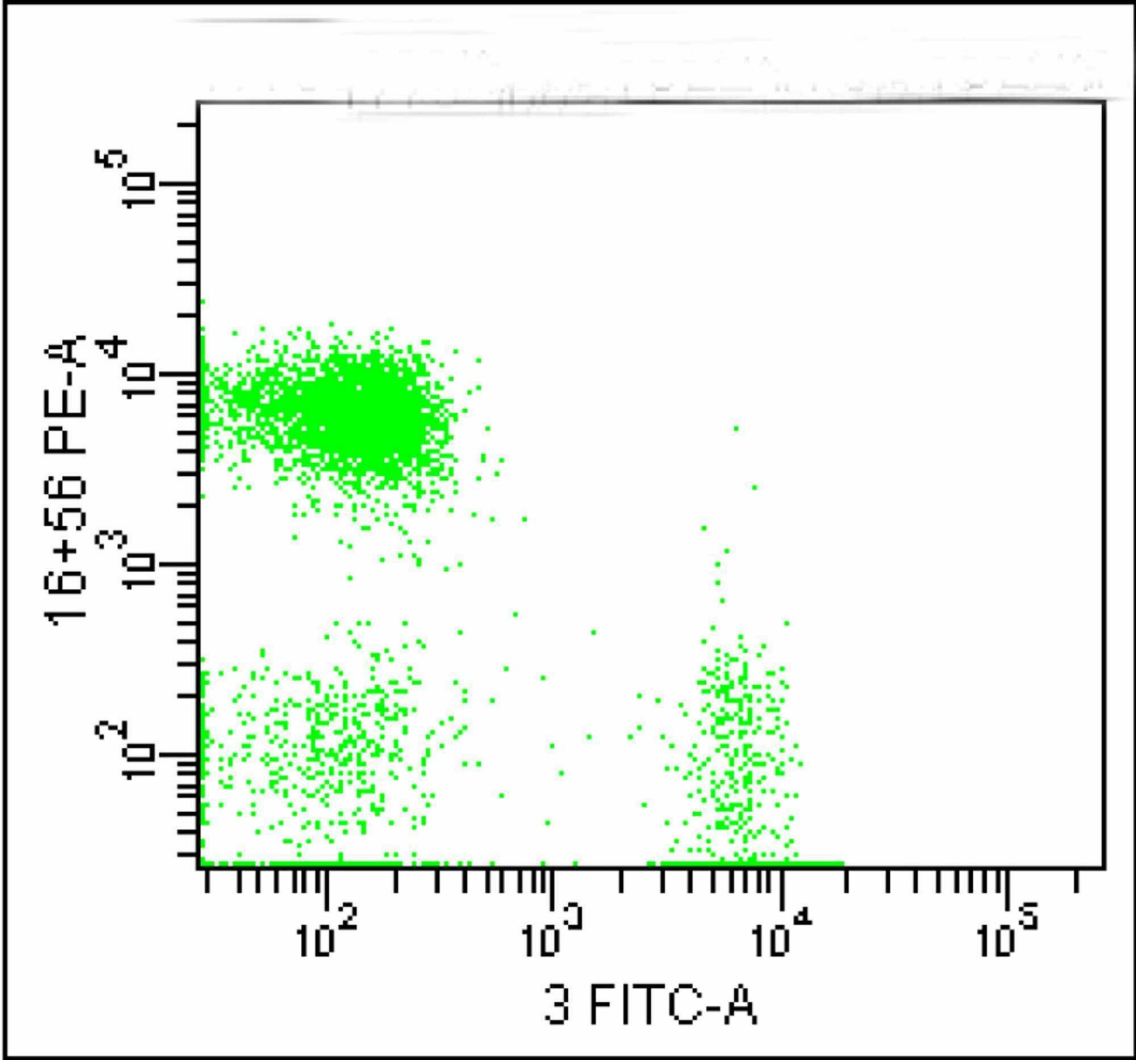
The dot plot shows a large population which is positive for CD16/56

On CD3 Vs CD16/56 gating the population of interest was positive for CD16/56 (Figure 6).

**Figure 7:**
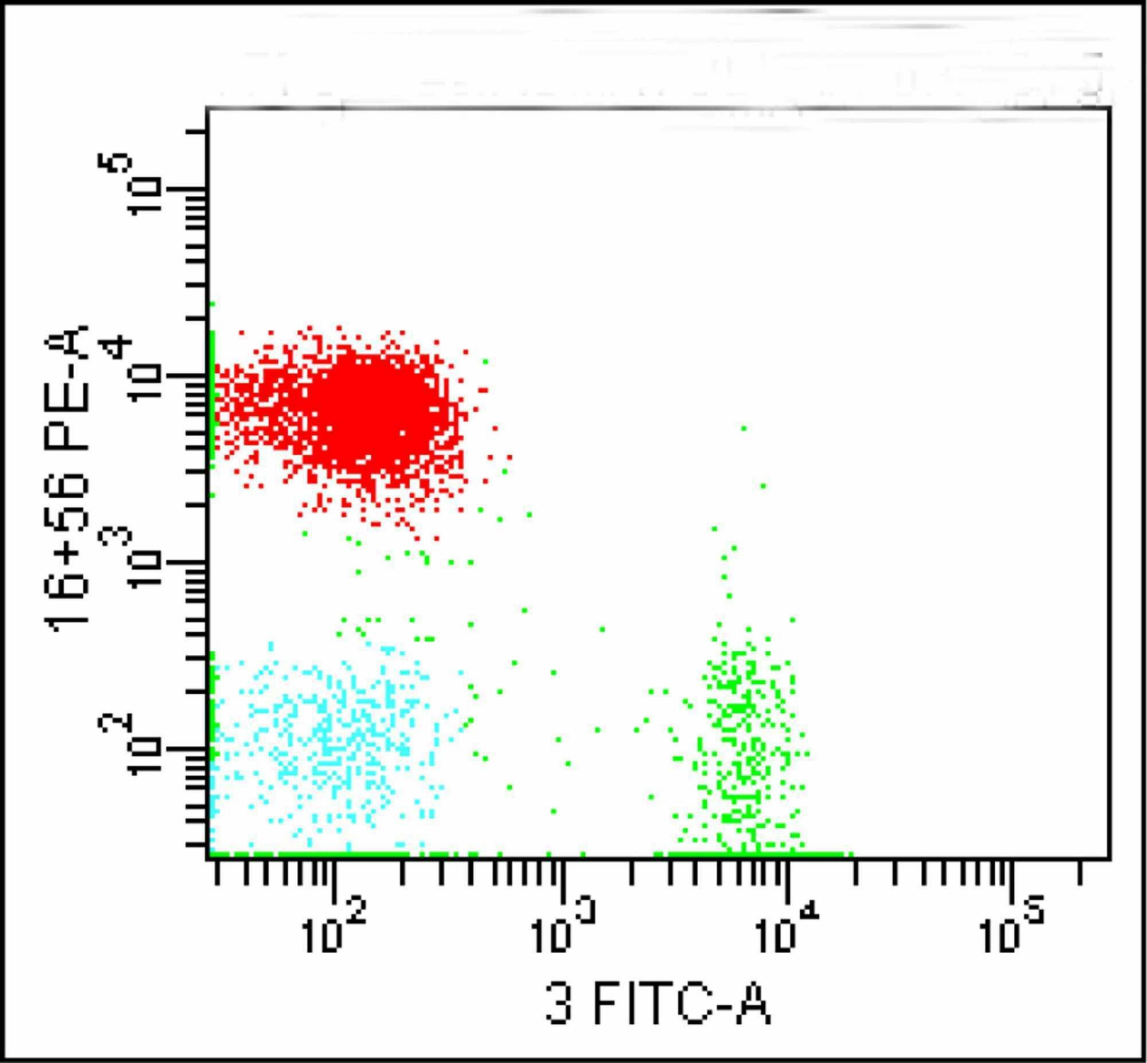
The CD16/56 population tagged in red colour

**Figure 8:**
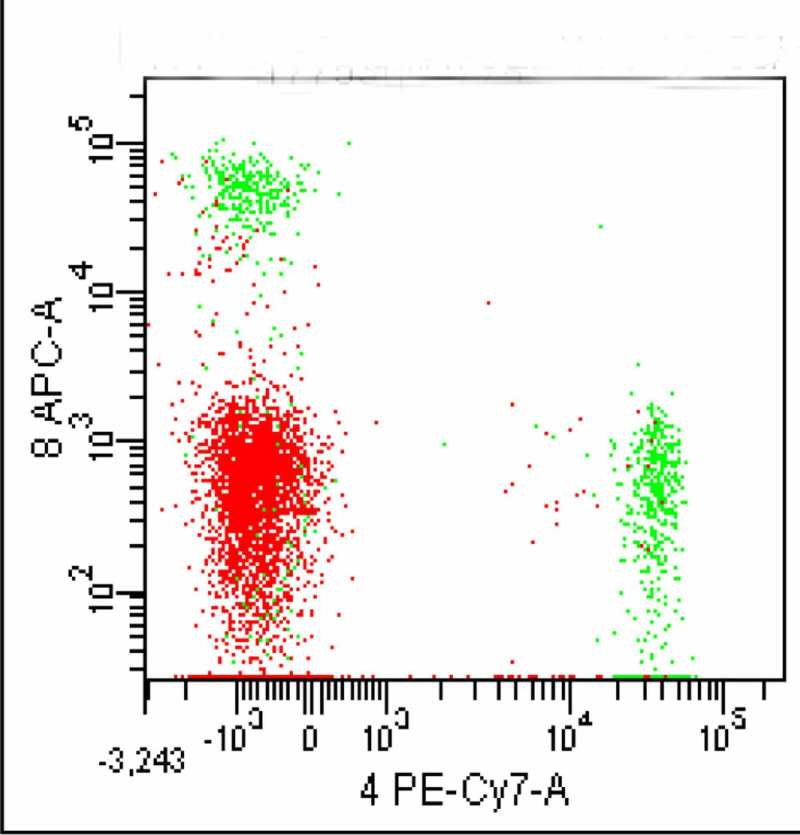
CD16/56 positive population (red color shows CD4 and CD8 negativity)

This population was tagged in red colour which later proved to be negative for CD4 and CD8 (Figures 7-8).

**Figure 9:**
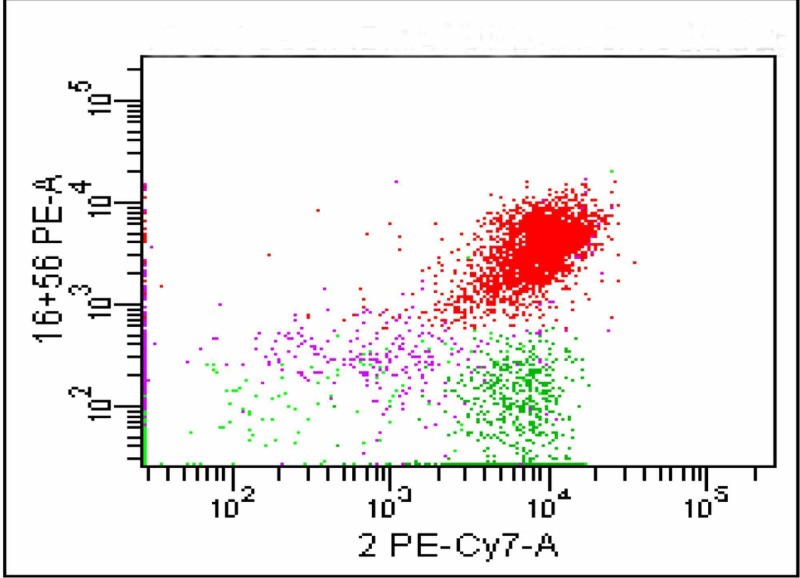
CD 16/56 positive population (red) is CD2 positive

**Figure 10:**
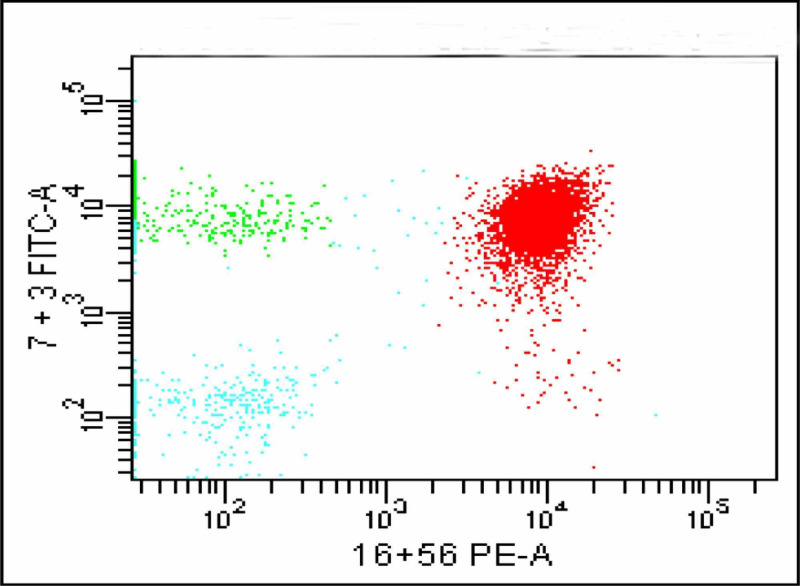
CD16/56 positive population is also CD7 positive

This CD16/56 positive population in red confirmed to be CD2 positive and CD7 positive (Figures 9-10).

Taking all this evidence into account, these CD45, CD16/56, CD2, and CD7 positive cells were confirmed to be NK cells. This NK cell population represented 43% of the total analyzed cells. This case was labeled as NK cell lymphocytosis, consistent with CLPD-NK.

## Discussion

Patients of CLPD-NK usually present with cytopenias, vasculitic skin lesions, non-neutropenic fever, recurrent neutropenic infections, musculoskeletal symptoms, peripheral neuropathy, aphthous ulcers, and splenomegaly. In contrast, our patient had no abnormality in any of the cell lineages. The only clinical manifestation which our patient had was recurrent pilonidal abscesses for which he sought medical advice. CLPD-NK is often associated with autoimmune diseases such as rheumatoid arthritis. Therefore, patients should be appropriately screened for all autoimmune diseases. However, in our patient, all autoimmune workup came out to be negative.

It is important to differentiate ANKL from CLPD-NK. Clinically aggressive natural killer leukemias will have an aggressive course with deterioration in the patient’s condition within a couple of months. On flow cytometric analysis, the former would have an abnormal phenotype, such as loss of CD16, CD56, or CD7 [5]. In our case, the patient’s clinical presentation and flow cytometric analysis were suggestive of CLPD-NK as NK cells showed no aberrant immunoprofile. Viral infection was further ruled out in this case as T lymphocyte count was not increased and the ratio of CD4 to CD8 positive T cells was maintained within the reference range.

According to a study done in 2012, signal transducer and activators of transcription-3 (STAT3) mutation is present in 30% of CLPD-NK cases. However, in our patient, mutational analysis was not done. Furthermore, CLPD-NK could not be categorized into one of its three subtypes as CD16 Vs CD56 gating was done [6]. Treatment of CLPD‐NK, which is only required in symptomatic cases, consists of immunosuppressive therapy including cyclophosphamide, methotrexate, cyclosporine A, and steroids [7]. 

## Conclusions

The diagnosis of CLPD-NK cells is quite challenging for pathologists owing to its rarity and similarity of NK cells to cytotoxic T cells. This disorder follows an indolent course which makes it even more cumbersome to detect it in initial stages but flow cytometry acts as a reliable diagnostic tool due to its high sensitivity. Furthermore, flow cytometric analysis plays an important role in differentiating CLPD-NK from other differential diagnoses which mimic a similar disease pattern. Treatment is only indicated in symptomatic patients. Hence, timely diagnosis is essential so that patients can be followed keenly for timely administration of appropriate immunosuppressive therapy as soon as the patient develops clinical manifestations.
